# Understanding progressive tissue loss and wound burden in combat casualties: lessons learnt for future operational capability

**DOI:** 10.1136/military-2022-002227

**Published:** 2023-11-22

**Authors:** Robert Staruch, D N Naumann, M Wordsworth, S Jeffery, R Rickard

**Affiliations:** 1 Department of Plastic & Reconstructive Surgery, Oxford University Hospitals NHS Foundation Trust, Oxford, UK; 2 Department of Engineering Science, University of Oxford, Oxford, UK; 3 Academic Department of Military Surgery and Trauma, Royal Centre for Defence Medicine, Birmingham, UK; 4 Department of Surgery, Queen Elizabeth Hospital Birmingham, Birmingham, UK; 5 Department of Burns and Plastic Surgery, Queen Elizabeth Hospital Birmingham, Birmingham, UK; 6 Department of Health Sciences, Aston University, Birmingham, UK

**Keywords:** Plastic & reconstructive surgery, WOUND MANAGEMENT, Trauma management

## Abstract

Understanding tissue loss following injury is important due to its prevalence among the war-wounded and the impact it has on subsequent treatment and rehabilitation. Progressive tissue loss is a type of tissue loss that has complicated extremity injury in recent conflicts. It has resulted in more proximal residual limb lengths and has influenced rehabilitation. Quantifying wound burden in combat casualties remains a challenge due to poor quality of data sets that lack the capacity for detailed analysis. The aims of this article are to outline the current hurdles in attempting to quantify wound burden in combat casualties and to propose simple interventions to improve data capture for future analysis.

**WHAT IS ALREADY KNOWN ON THIS TOPIC**

- Progressive tissue necrosis (PTN) is a phenomenon observed in the management of blast-related combat casualties from operations in Afghanistan.
- Although frequently referenced in historical accounts of surgical care, little quantifiable data are available to understand PTN.

**WHAT THIS STUDY ADDS**

- Collecting retrospective data to quantify PTN is complex and is unable to give clarity on the causative factors of this clinical condition.
- Surgeons should proactively quantify wound burden as a marker of disease progression, such as seen in burn injury.

**HOW THIS STUDY MIGHT AFFECT RESEARCH, PRACTICE OR POLICY**

- To understand future wounds in combat casualties, this paper postulates several simple measures to facilitate understanding of wound burden on the patient and its potential evolution.

## Introduction

 Tissue loss following injury is important because of its prevalence among the war-wounded and the impact it has on subsequent treatment and rehabilitation. Tissue loss observed from major trauma inflicted from large explosions in recent conflicts has been termed ‘progressive tissue necrosis’ (PTN).^1^ This is defined as the loss of soft tissues such as skin, fat, fascia and muscle over the subsequent 7–10 days from injury despite multiple trips to the operating theatre to debride tissues. This may have contributed to more proximal leg lengths on hospital discharge. However, necrosis is thought to be only one of the causes of such tissue loss, and therefore in the current article PTN will be referred to as progressive tissue loss (PTL) to prevent confusion on the potential mechanisms underlying this condition (such as necrosis, necroptosis or apoptosis). Some of the aetiological factors may include dysregulation of the systemic inflammatory response,^2^ microvascular endotheliopathy of trauma,^3^ invasive infection from tissue contamination^4^ or a mechanobiologically initiated cellular injury.^5^ Some, or all, of these may contribute to a tissue environment that is not compatible with cell survival. However, the precise causes and the relative contribution of each have not yet been elucidated.

Patients with major trauma and burns are often treated as different patient groups with areas of overlapping physiology. Clinicians have alluded to the similarities between combat casualties and burn patients. Nevertheless, the physiological principles that underpin burn care—that skin loss and soft tissue integrity drive the larger systemic response and its recovery—have not materialised into better wound documentation. Regular documentation of wound metrics and its adoption in the characterisation of combat casualties could be a useful paradigm change.

### Aims and focus

The aims of the current article are to describe the challenges of quantifying tissue loss in combat casualties and to present potential solutions in wound documentation to facilitate future analysis in civilian and deployed trauma settings.

## Defining the problem space and the current challenges in quantifying tissue loss

Although observational accounts exist, there is currently no quantifiable evidence to suggest PTL existed in combat casualties, and more precisely what drove it. Analysis of operative records from deployment has demonstrated that plastic surgeons were required in over 40% of cases in Camp Bastion, potentially illustrating the potential burden wounds may have in these trauma patients.^6^ However, detailed data on wound size, depth and burden are lacking.

To probe what potentially drove this phenomenon (such as mucormycosis^7^ or programmed cell death), we must first be able to demonstrate it quantifiably existed. Historically, combat casualties have been characterised by their injury severity score,^8^ or grouped by the number and level of amputations (single, double or triple amputee).^9^ As PTL has not been characterised in this patient group, it is uncertain to what extent PTL influenced a patient’s reconstruction, such as residual limb length, or rehabilitation, such as mobility in prosthetics. More fundamentally, we have no system for identifying these patients as a clinical group, such as one might do if they were a burn (superficial vs partial thickness). There have been attempts to demonstrate PTL through conducting subgroup analysis on severely injured casualties, such as the ‘survivable unsurvivables’, which in itself has drawbacks. The main drawback is that this approach misses out patients who may have had large soft tissue wounds as their primary injury alone that necessitated reconstruction but not amputation.

In order to quantify PTL specifically, clinicians would need comprehensive data on patients’ wound, their physiological status and their reconstructive history. These may include serial data points for wound size or surface area taken from standardised intraoperative photographs or documentation. Furthermore, illustrations of the tissue types involved will be necessary. Perhaps these images could then be cross-referenced against radiographs demonstrating the change in bony length over time, although this would need a cross-specialty consensus and validation of the methodology. Wound evolution data could also be evaluated alongside markers of soft tissue injury, such as creatine kinase (CK), infection (wound microbiology) or evidence of sepsis (if validated). This potentially would allow a robust assessment of wound progression over time and aid in understanding potential clinical causative factors.

Adapting existing databases to store wound images therefore is the first challenge. The UK and the USA both employ the Joint Theatre Trauma Registry (JTTR) to document deployed combat and non-combat injury, and this records mechanistic, physiological, diagnostic, therapeutic and outcome data. As an example, while the JTTR records data on wound mechanism, it does not record the size or depth of a patient’s wound. As such, identifying subsets of patients based on their wound size or type is difficult, unless JTTR records can be linked to domestic hospital records to review surgical notes (such as units as Walter Reed in the USA or the Royal Centre for Defence Medicine (RCDM) in the UK). Data sets that precisely define the wound alongside key patient data, such as the International Burn Injury Database, may be models for collecting future data during operations, whether as an adjunct to the JTTR or as a separate system. The system for attaching wound information to electronic patient records should be improved. This conundrum is not isolated to the military since deployed civilian organisations treating acute traumatic injuries face similar problems when attempting to move patients into their host nation’s healthcare system or transfer them elsewhere. Most importantly, any future system must interact with patient records to allow seamless patient care.

The second challenge is adopting uniform terminology to document and describe wounds between clinicians. Standardised language has been adopted to describe open lower limb fractures, such as the Gustilo Anderson classification, as well as burn injuries, yet there remains no common terminology to describe large soft tissue defects from trauma. Historically, the Red Cross system for wound documentation has been the ‘gold’ standard for documentation of traumatic wounds at the time of primary surgical debridement.^10^ However, clinicians report this system as difficult to remember and cumbersome to document, particularly when overwhelmed by patient volume. Ideally, clinicians require a straightforward set of metrics that can be employed at arm’s reach or easily recalled from memory to demonstrate wound injury burden and their healing (or evolution) over time. Therefore, establishing a simple standardised method for describing wounds is a critical piece of work.

Wound photographs are commonly used in civilian practice to inform clinical decision making and are routinely attached to patient electronic records. This is a significant technological gap in defence that is potentially inhibiting the optimal management of combat casualty wounds.

Although clinicians historically took serial photographs of wounds from casualties during debridement in recent conflicts, particularly at the RCDM, the methodology for this has not been standardised. These were taken to inform decision making at multidisciplinary team (MDT) meetings, rather than as a quantitative marker of tissue injury.

As such, the views obtained, the camera set-up (zoom, distance from subject, lighting, etc) and the use of objects for scale bars (rulers in view for scale) are lacking from these images. Thus, comparing changes between serial images is impossible unless there is a degree of scaling or standardisation across these images. More broadly, collating wound images among all injuries would enable a better understanding of the entire spectrum of combat wounds, not just the most severe. This data set is currently lacking and this is limiting our understanding of these patients. Hence, bridging this gap is an important learning outcome from previous conflicts that would avoid a ‘walker dip’.

First, it limits our understanding of how large combat wounds are and how they evolve. Furthermore, it blunts our understanding of when they transition from a wound in evolution to a stable wound ready for reconstruction. It also prevents us from understanding their burden of care—in terms of how they were managed both in time, physical resources and the patient’s gross physiology at the time. Finally, it inhibits our ability as clinicians to influence and improve our patients’ care as we are unable to know what factors optimise wound outcomes, slow progression or improve healing in this patient group. Such a data set would allow us to correlate wound progression with patients’ physiology or laboratory results, giving insight into how complex wounds may influence systemic inflammation after major trauma. There are several key research questions that also remain unanswered beyond characterising PTL. How do the mechanism and the size of the wound influence the systemic response in these patients? How did the wound influence the choice of reconstruction and subsequent rehabilitation? Although the ADVANCE study may give us insight into this latter area, the lack of combat wound data will make it challenging to draw causality between these three areas. The care delivered during Op HERRICK is touted as a once-in-a-generation model; however its central ethos—the delivery of the highest quality of care possible—should remain the central theme of future approaches to the surgical reconstruction of patients with PTL. Hence, the data collection and the characterisation of wound burden in our patients are the baseline standards of care our patients should expect.

## Solutions for quantifying wound burden in future conflicts

As discussed, quantifying PTL from operations is a challenge but will be an adjunct for improving patient care. This remains an important sophistication gap that should be bridged to enable the delivery of optimal patient care. Here we will discuss some interventions that might be adopted to aid quantification of wound burden in trauma.

### Primary wound assessment

Deployed healthcare professionals dealing with major trauma should establish a simple and standardised protocol for wound assessment and record keeping both on deployed operations and in home nations. This should include a standard list of information recorded at initial debridement (and subsequent debridements) for record keeping of the wound, in particular wound depth, anatomical location and constituent components. The total wound surface area, similar to that used in burn injuries, should also be documented. These wound metrics should be recorded regularly at surgical debridement and reviewed at regular MDT meetings where the wounds can be evaluated in the context of other ongoing sequelae of traumatic injury (such as inflammation, infection, etc). It is important to clarify that while some of this work has already been undertaken (wound photography and evaluation at an MDT with a view to closure), these have been subjective rather than objective assessments. Nevertheless, the development of a robust wound assessment tool for acute traumatic wounds would be a useful basic clinical adjunct.

### Wound imaging

The use of wound photographs over time plays an important role in wound evaluation and documenting wound evolution. Much has been written concerning the validity of observing clinical features from wound photography and reducing interuser variability.^11^,^13^ Recently, machine learning approaches have been adopted to automate the diagnosis of surgical site infection in emergency surgery patients.^14^

To use wound images to evaluate burden, the type of images (the views), lighting and scaling must be standardised. Such standardisation has been demonstrated in published randomised controlled trials of wound photography in trauma. Limitations such as using digital zoom must be well known to the user population to avoid documentation error. Table 1 outlines some of the features that should be employed to achieve this. Potentially, if images are to be used to assess wound depth and tissue quality, then lighting and the use of flash must be considered (eg, removing operative lights when taking the shot). The use of mobile technology and encrypted messaging services in healthcare means that high-quality images can be obtained at the bedside in even the most austere environment. In one evaluation of images captured using camera phones, it was demonstrated that the images could be analysed to capture wound size and depth irrespective of light conditions (as this could be adjusted for in analysis).^15 16^ Nevertheless, the clinician behind the lens should be aware of the simple steps to ensure the photographs are of use for wound evaluation. Similar to bony radiographs, one image should include the anatomical region above and below the wound for orientation, with an indicator of the cranial and caudal end of the patient. Furthermore, an object of known length should be in view to allow for scaling. Subsequent more detailed photos of the wound bed can then be taken and viewed in the context of the initial orientation image.

**Table 1:** Suggested good practice protocol for documenting combat wounds

Feature	Explanation
Set-up	Camera, zoom used and estimated distance from the patient.
Scale	Has a standardised object of known distance been included in the image—sterile ruler? Bic Biro?
Image set	What set of images has been obtained to orientate the viewer to the wound position/location and size? Are these numbered within the shot?
Image labelling	Has tissue of different viability been indicated to enable subsequent colour appreciation/analysis? That is, has dark tissue been labelled as dead so viewers do not confuse potential light effects with tissue effects?
Image lighting	Has the operative light been removed from the field for the photograph? Is the photographer using flash?

### Wound quantification

#### Wound size

**Figure 1:**
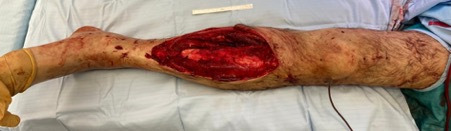
Example image with scale bar and appropriate lighting. Image includes the joint above and below the wound.

**Figure 2:**
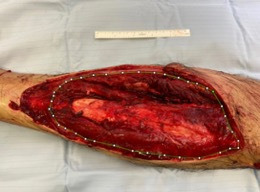
ImageJ software was used to analyse the figure and estimate the wound surface area. Wound per cent is calculated by estimating the wound surface area as the percentage of the limb surface area visible on photography.

**Figure 3:**
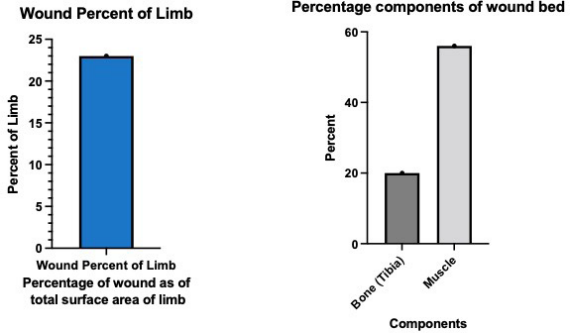
Percentage of the components of the wound bed. The surface area tool in ImageJ was used to assess the total area of the wound image occupied by different tissues.

Wound size has been directly correlated with mortality in burn injuries.^17^ The physiological response to severe traumatic injury, such as blast injuries, is similar to that seen in major burns. Yet little work has been done in this patient group to demonstrate causality between wound sizes and mortality. As alluded to, part of this problem is due to a lack of coherent data. Hence, an effort should be made to quantify wound size and characteristics in the acute injury phase. This includes estimating the wound surface area of the patient, the techniques for which have been described in the literature.^18 19^ figure 1 and figure 2 demonstrate how suface area estimation can be applied to wound photos using IMAGE J software. Figure 3 demonstrates how surface area data can be used to evaluate wound composition and proportions. One drawback of assessing wound surface area lies in the confounding effect of amputations, where patients have lost a significant volume of tissue, yet resultantly have relatively small wounds afterwards. In such instances, techniques that assess change in body surface area may be more accurate methods to characterise the effect of wound size on patient health.

Efforts should also be made to clarify the constituent components of the wound and any indications of early signs of infection or extensive fluid losses. Techniques for this could include recording the frequency of dressing changes or the volume of fluid collected over time in a negative pressure dressing.

Within defence, Pando is revolutionising reach forward capability within the Defence Medical Services.^20^,^22^ This encrypted messaging service has enabled multidiscipline discussion of patients and challenging cases between deployed and firm base clinicians. Furthermore, it provides a secure and fast means to access advice or clinical consensus. It has also enabled the communication of patient images to facilitate discussion.

**Figure 4:**
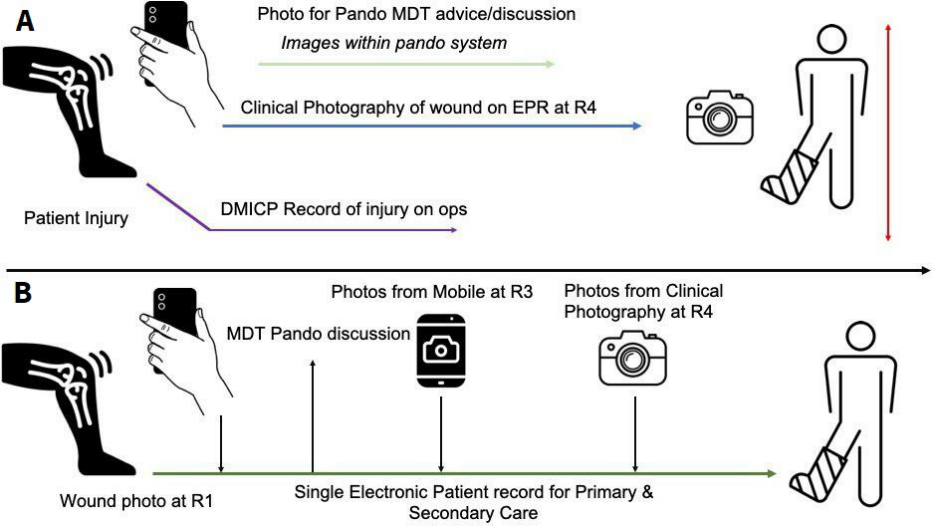
(A) Outline of current pitfalls of image documentation in defence patients. (B) How a streamlined electronic patient record could facilitate wound data collection and MDT discussion. MDT, multidisciplinary team. R3, Role 3; R4, Role 4.

The technology gap in this area is the bridge between information sent within Pando and the patient’s medical records (held within the Defence Medical Information Capability Programme - DMICP). In civilian systems, such as the Cerner’s electronic patient record system, clinicians are able to take patient photos (such as of wounds) and directly upload them onto the patient record. This is enabled through a mobile app version of the computer Electronic Patient Record (EPR) software. This allows them to be viewed by other clinicians and also provides a central location (attached to the patient record). This capability is currently not available in the DMICP, nor is a link between the DMICP and patients’ subsequent medical records at role 3 or 4. A unified electronic patient record would overcome this hurdle, particularly if both deployed and firm base users were able to upload and view images. This would clearly improve patient care. An alternative approach would be to build a bridge between Pando and soldiers’ healthcare records—both at role 4 (civilian patient records) and DMICP—so that such images are not lost. figure 4 summarises the current state of play and the potential direction to bridge this sophistication gap.

Regardless of the system adopted, the formal consideration of wound size in the context of patients’ ongoing state is important in being able to comprehend whether they are deteriorating or healing. While there is no question that such considerations have to date not been featured as part of routine clinical care in trauma, quantitative data are lacking.

#### Weight of tissue

One technique used in some surgical disciplines, such as breast surgery, is to weigh the excised tissue. This allows surgeons to compare excised breast tissue between the two sides and also to assess whether further excision (in some cases) is necessary. It also allows them to get an understanding of likely volume symmetry. The tissue is weighed intraoperatively and recorded in the operative notes. Logistically, this is a simple and straightforward intervention to introduce, as only a set of weighing scales are required to weigh intraoperative tissue. The total excised tissue can then be photographed on a surgical drape, for record keeping in the notes, along with the total tissue weight. Recording of tissue weight would be a useful adjunct for assessing the burden of debridement. Although it would be difficult to correlate excised tissue with overall body mass, evaluating the trend of tissue weight over multiple excisions could be of clinical use. A downward trend (a reduction in excised tissue) may demonstrate an improvement in wound condition or point towards the wound being free of infection. Finally, records of tissue debridement weight over time would facilitate the quantification of the presence of a PTL.

### Wound biomarkers

If patients’ clinical data (blood tests, radiographs and wound images) were joined seamlessly throughout their journey, the next question is what markers are the best adjuncts for assessing soft tissue health and loss. Markers of infection, such as white cell count or C reactive protein, are indeterminant markers of soft tissue health. Enzymes such as CK are known markers of skeletal muscle injury, but perhaps not sensitive enough in larger wounds. These markers may all become less sensitive in the context of major trauma. Results may be heavily influenced by the initial injury, making the interpretation of serial measurements more challenging. As such, a more detailed analysis of circulating markers should be undertaken to assess which is the most sensitive and specific in major trauma.

A biomarker, as defined by the National Institute of Health Research, is ‘a characteristic that is objectively measured and evaluated as an indicator of normal biological processes, pathogenic processes, or pharmacologic responses to a therapeutic intervention’*.*^23^ More recently, biomarkers have been postulated as being categorised as predictive, diagnostic or indicative based on the information they provide. Specifically to wound healing, biomarkers have been extensively examined as an adjunct to the progression of chronic wounds.^24^ However, little has been undertaken to identify potential markers in acute wounds, or more specifically acute traumatic wounds. In addition, there exists no biomarker capable of characterising injury to specific soft tissue components, such as muscles over fat or skin. Markers such as CK and troponin can be used to corroborate known muscle injury, and large values can give some indication of the size of the skeletal muscle injury. However, neither of these is sensitive enough to chart an ongoing progressive muscle loss. As specific markers of muscle or fat remain elusive, it is therefore difficult to assess the severity of injury for these tissue components over time.

In the discipline of burn care, several laboratory-based systems correlate burn injury severity with circulating markers. The prognostic inflammatory and nutritional index that uses albumin, prealbumin, orosomucoid and C reactive protein correlates these markers with burn injury. Furthermore, changes in renal function have also been correlated with burn mortality, as well as base deficit and lactate change over time. Inflammatory markers such as TNF-alpha and the cytokines IL-6, IL-8 and IL-10 have also been correlated with burn mortality.

One initial approach may be to assess published laboratory scoring systems in combat casualties to evaluate sensitivity. The drawback of this approach is the need to collect good-quality prospective combat casualty patient data. This presents its own challenges.

## Future directions

Recent deployed experience reminded the military MDT of the nature and severity of combat war wounds and their major contribution to patients’ care burden. The lessons learnt from this experience need to be translated into practical interventions to facilitate proactive wound care being an important part of a patient’s acute care, rather than an afterthought in a patient’s clinical journey. The resurgence in conflict in Europe palpably reminds us that combat casualties are an ever-present patient population. Improving best practice in documenting and treating these patients is therefore a multidisciplinary ongoing effort of military and civilian clinicians. Much of our clinical decision making in combat wounds has been derived from evidence and knowledge in burn injury, as the former group demonstrates physiological similarities to patients with severe burn injuries. However, our focus should be building an evidence base for this group of patients to underpin our clinical decision making. As argued in this article, one of the current problems in this space is the lack of a robust database of standardised images of combat wounds that would enable robust analysis. This would allow clinicians to understand this injury burden in the context of others reported in the literature. Accurate wound patient data are therefore useful in guiding the overall injury pattern seen, how it should be managed, by whom and in what resource. These last two questions are particularly critical to many military organisations where defining the optimal forward surgical team is an important ongoing debate.

To this end, there are three next steps in the journey to improve combat casualty wound metrics. These are listed in their ease of implementation. The first is to agree and adopt the suggested techniques to photograph and document wounds at the bedside level by treating clinicians. This is particularly important from initial care at role 3 to discharge at role 4 (or rehabilitation facility). To achieve interoperability of wound assessment between nations, we must first develop a robust system that is simple but effective for our clinicians. The second step is to investigate how to collate these wound images into an accessible database that could be used for analysis. This second step may be nation-specific and will involve adapting or expanding existing defence technology (such as Pando or electronic health records). Finally, the third step is to engage with industry and academia to understand potential wound biomarkers in traumatic wounds through targeted funding and research collaboration. This last step will require important discussions about how we collaborate to collect data from combat casualties in future conflict zones.
